# Postnatal Outcome and Associated Anomalies of Prenatally Diagnosed Right Aortic Arch with Concomitant Right Ductal Arch: A Systematic Review and Meta-Analysis

**DOI:** 10.3390/diagnostics10100831

**Published:** 2020-10-15

**Authors:** Paolo Ivo Cavoretto, Alexandros Sotiriadis, Serena Girardelli, Silvia Spinillo, Massimo Candiani, Silvia Amodeo, Antonio Farina, Vlasta Fesslova

**Affiliations:** 1 Obstetrics and Gynecology Department, IRCCS San Raffaele Hospital, University Vita-Salute, 20132 Milan, Italy; girardelli.serena@hsr.it (S.G.); spinillo.silvia@hsr.it (S.S.); candiani.massimo@hsr.it (M.C.); 2 Second Department of Obstetrics and Gynecology, Faculty of Medicine, Aristotle University of Thessaloniki, 54124 Thessaloniki, Greece; asotir@gmail.com; 3 Division of Obstetrics and Prenatal Medicine, Department of Medicine and Surgery (DIMEC), Sant’Orsola-Malpighi Hospital, University of Bologna, 40138 Bologna, Italy; silviaamodeo23@gmail.com (S.A.); antonio.farina@unibo.it (A.F.); 4 Center of Fetal Cardiology, IRCCS Policlinico San Donato, 20097 San Donato Milanese, Milan, Italy; Vlasta.Fesslova@grupposandonato.it

**Keywords:** right aortic arch, right ductal arch, congenital heart defect, 22q11 microdeletion

## Abstract

Right aortic arch presents a reported incidence of 0.1% of the general population; the aim of our study was to evaluate the risk of associated intracardiac (ICA), extracardiac (ECA), or chromosomal abnormalities in fetuses with right aortic arch (RAA) and concomitant right ductal arch (RDA). A systematic review of the literature selected 18 studies including 60 cases of RAA/RDA. A meta-analysis with a random effect model calculated for each outcome the pooled crude proportion of associated abnormal outcomes in cases of RAA/RDA and the pooled proportions and odds ratios in RAA with LDA or RDA. Quality assessment of the included studies was achieved using the NIH quality assessment tool for case series studies. RAA/RDA presents risk of associated conotruncal CHDs of about 30% and risk of 22q11 microdeletion in the region of 1%. Two-thirds of 22q11 microdeletions had concomitant thymic hypoplasia and no other chromosomal defects were described. Risks for ICA, ECA, 22q11 microdeletion, and aberrant left subclavian artery are not substantially different in RAA with right or left arterial duct. RAA increases the risk of associated cardiac defects regardless of laterality of the ductal arch. In isolated RDA/RAA cases, absolute risks of extracardiac associated problems or surgery are rather low, we would therefore recommend reassurance, particularly when the thymus and karyotype are normal.

## 1. Introduction

### 1.1. Rationale

The prevalence of aortic arch anomalies, including right aortic arch and double aortic arch is estimated to be approximately 0.1% in the adult population and low-risk fetuses [1,2]. Right aortic arch (RAA) is characterized by abnormal laterality of the aorta and the brachiocephalic vessels. RAA courses to the right of the trachea, in contrast to the normal left aortic arch (LAA), as a result of abnormal regression of the primordial aortic arch system during embryogenesis [3].

Classification of congenital aortic arch abnormalities in four groups was reported decades ago: group I includes double aortic arch, group II left aortic arch, group III right aortic arch, and group IV other and rare malformations of the aortic arch system. Recently this classification wan challenged and enriched with several subgroups, in order to incorporate more recent definitions [2].

RAA is diagnosed using both two-dimensional gray-scale ultrasound and color Doppler imaging, when the transverse arch is imaged to the right of the trachea on axial views of the fetal chest, at the level of the three-vessel and trachea view [4]. In fact, current International Society of Ultrasound in Obstetrics and Gynecology (ISUOG) practice guidelines for sonographic screening examination of the fetal heart recommend the three-vessel and trachea view to be included in routine pregnancy screening in order to increase prenatal detection of many cardiac and vascular abnormalities, including RAA [5].

The majority of fetuses with an isolated RAA present a left-sided ductus arteriosus (LDA) while a right-sided arterial duct (RDA) is identified in approximately 10–15% of cases of RAA [6,7,8,9].

The LDA always enters the RAA from the left side encircling the trachea and creating a U-shape (U-sign), while the RDA lies to the right of the trachea, parallel to the right-sided aorta, forming a V-shape (V-sign) [10].

RAA may be present with mirror image branching of a normal left aortic arch, giving rise to the left innominate, right carotid, and right subclavian arteries in sequence. Nevertheless, RAA with aberrant retroesophageal/retrotracheal left subclavian artery (ALSA) forming a vascular ring is also common [11]. RDA was described in about 10% of cases with RAA [7]. ALSA has been reported in up to 40% of fetuses with a right-sided aortic arch; however, its association with concomitant right arch and duct is less clear [11].

RAA detected in fetal life is frequently associated with other intra-cardiac (ICA) and extra-cardiac anomalies (ECA) [11,12,13]. The association between RAA and ICA, ECA, or chromosomal abnormalities (particularly 22q11.2 microdeletion) was described before; however, a clear risk quantification was not carried out [14].

Postnatal prognosis of patients with RAA and congenital heart defects is usually determined by the severity of the associated anomalies, particularly ICA [13]. Conversely, the prognosis of fetuses with RAA and normal cardiac anatomy is determined by potential vascular compression of the trachea or esophagus, giving rise to respiratory symptoms or dysphagia. With high definition ultrasound and increasing expertise in fetal ultrasound, prenatal detection rate of aortic arch anomalies is increasing. However, little is currently known about how to counsel patients regarding fetal and neonatal prognosis when right aortic arch coexists with right arterial duct.

### 1.2. Objectives

The primary aim of our study is to evaluate the risk of associated ICA, ECA or chromosomal abnormalities in fetuses prenatally diagnosed with RAA and concomitant RDA, in order to optimize prenatal counseling and perinatal management. The secondary objective is to compare the described outcomes in fetuses with RAA and LDA to those with RAA and RDA. 

## 2. Materials and Methods

### 2.1. Search Strategy, Information Sources and Eligibility

The MEDLINE and EMBASE databases were searched using the following keywords alone or in different combinations: “congenital heart defects,” “right aortic arch,” “prenatal,” “right ductus arteriosus,” “ultrasound,” “fetal,” “right ductal arch,” “prenatal diagnosis,” “ultrasound,” and pregnancy.” Search was limited to articles published in English language. This work is presented in agreement with PRISMA guidelines [15].

Only studies reporting prenatal diagnosis of RAA with concomitant RDA in the three vessel and trachea view were collected, including an unpublished case from our personal experience. Retrospective, cohort, longitudinal studies, and case reports were included. Pediatric and surgical postnatal series were excluded as they are based on a different population with a potential selection bias. 

### 2.2. Study Selection, Data Collection, and Outcomes

**Figure 1:**
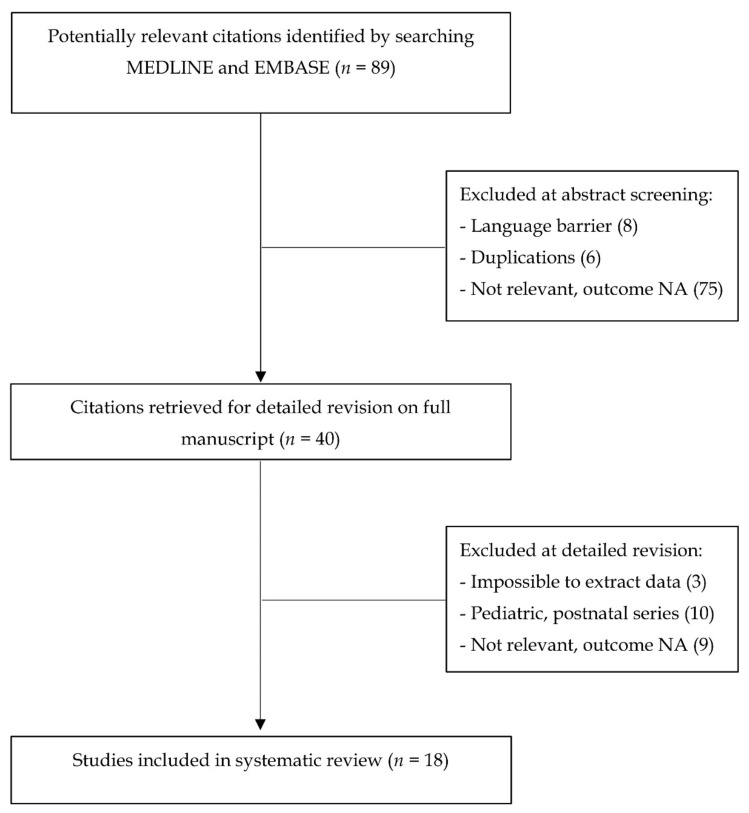
Flow chart of study design. NA: not available.

Flowchart of study selection is shown in Figure 1 [15].

Data concerning the neonatal outcomes of fetuses prenatally diagnosed with RAA with RDA were collected and recorded in a dedicated database by two different authors. Data concerning neonatal outcome of fetuses prenatally diagnosed with RAA from studies clearly describing both subgroups with RAA and LDA were also collected and recorded separately, by two different authors. 

The outcomes analyzed were chromosomal defects (CD), intracardiac or vascular abnormalities (ICA), extracardiac abnormalities (ECA), fetal growth restriction (FGR), termination of pregnancy (TOP), gestational age at prenatal diagnosis, neonatal features, respiratory symptoms, intrauterine demise (IUD) and cardiac surgery. Two authors (S.G. and S.S.) reviewed all articles independently and consensus was reached about relevance and inconsistencies. Any doubt and inconsistency was resolved by consulting senior authors (P.C., A.S., A.F., V.F.). The study did not require ethical approval. 

### 2.3. Study Quality Assessment

Quality assessment of the included studies was achieved using the National Institute of Health (NIH) tool for the quality assessment of Case Series Studies (https://www.nhlbi.nih.gov/health-topics/study-quality-assessment-tools. Access). This method was also recommended by the National Institute for Health and Care Excellence (NICE). The grades were attributed based on questions 1–9: “good” if questions 1, 6, and 7 (principal factors) were present; “fair” if two factors were present; and “poor” or “insufficient quality” if one factor was present. A global assessment (good, fair, and poor) according to Agency for Healthcare Research and Quality (AHQR), NICE and NIH standards was assigned to each study.

### 2.4. Statistical Analysis

The crude proportions of associated abnormal outcomes described above were calculated combining events occurring in cases of RAA/RDA collected from all the selected studies and are presented as percentages with 95% confidence interval.

Studies reporting on both RAA/RDA and RAA/LDA were used to assess the outcomes of interest according to the laterality of the ductal arch (RDA or LDA), after creating contingency tables with raw data, which were used for meta-analysis. 

For each outcome, the pooled proportions in each group (RAA/LDA and RAA/RDA) were calculated from all available studies, using the metaprop package on Stata. Then, the frequency of the outcomes in the two groups was compared using data from studies reporting on both RAA/RDA and RAA/LDA fetuses. The comparisons between the two groups were performed using odds ratios with 95%CIs. Because of the anticipated clinical heterogeneity, a random effects model was used, except for outcomes with zero events in one of the groups. In the latter case, Mantel–Haenzsel fixed-effect OR was calculated, which incorporates evidence from single-zero studies without requiring continuity corrections [16]. Studies with zero events in both arms were excluded from the meta-analysis. The significance of the combined OR calculated using the Mantel–Haenszel statistical method was determined by the Z test and the *p* value. To assess the between-study heterogeneity, we used the I^2^ statistic. The I^2^ index expresses the percentage of the total variation across studies that is due to heterogeneity. I^2^ values of 25, 50, and 75% correspond to low, moderate, and high heterogeneity, respectively [17,18]. Forest-plots were used to display proportions and ORs. Pooled ORs are represented in a logarithmic scale. Quantitative assessment of publication bias with funnel plots was performed when appropriate [17]. Statistical analyses were performed using Review Manager version 5.3 (RevMan, The Nordic Cochrane Centre, The Cochrane Collaboration, 2014, Copenhagen, Denmark) Open Meta-Analyst (http://www.cebm.brown.edu/openmeta/index.html) and Stata (Stata 15.1, StataCorp LP, College Station, TX, USA). 

## 3. Results

### 3.1. Study Selection and Characteristics

Eighteen articles were included, with sample size ranging between 1 and 10. An overall number of 60 fetuses diagnosed with RRA and RDA was found. A personal case from our records at the Fetal Medicine Unit of San Raffaele Hospital and recently presented at ISUOG Congress was added [19]. Sixty percent of cases were diagnosed before 22 gestational weeks (range 11–36).

### 3.2. Synthesis of Results

#### 3.2.1. Primary Analysis: Proportions of Abnormal Outcomes in RAA/RDA

**Figure 2:**
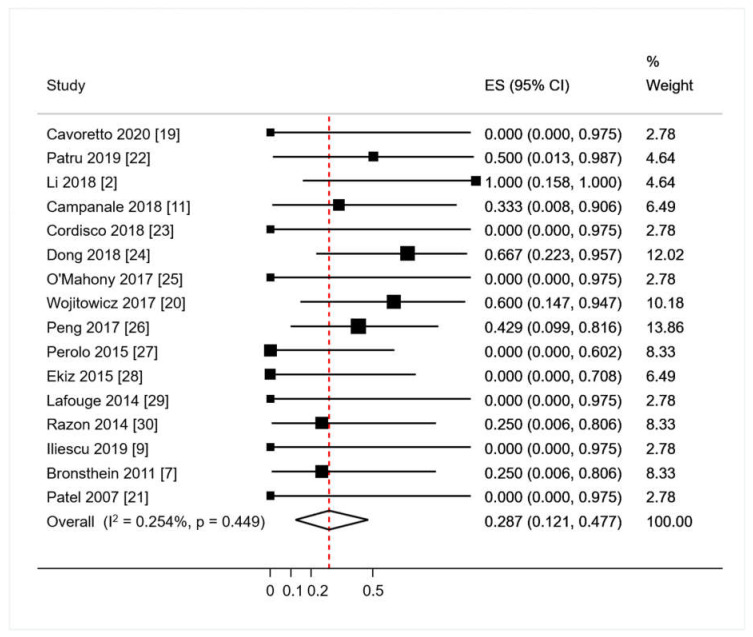
Pooled proportion of intracardiac anomalies in RAA/RDA (right aortic arch with concomitant right ductal arch) fetuses. ES: effect size. CI: confidence interval. Red dashed line describes the effect estimate found in the present analysis.

Live birth occurred in 52/53 (98.1%); termination of pregnancy in 1/53 (1.9%). Fetal growth restriction was described in one study, [20] occurred in 3 of the 52 liveborn (5.7%) and no IUD or NND were recorded. Forty-six patients from studies encompassing both isolated RAA/RDA and fetuses with both RAA/RDA and major ICAs were registered; associated ICAs were present in 16/46 of RAA/RDA fetuses (raw proportion 34.7%). All ICAs were conotruncal anomalies: three tetralogies of Fallot (TOF) (6.5%); two double outlet right ventricles (DORV) (4.3%); five pulmonary artery abnormalities 5 (10.8%); two transpositions of the great arteries (TGA) (4.3%), a truncus arteriosus communis (2.2%), a persistent left-superior vena cava, a pulmonary atresia with ventricular septal defect, and three cases of not-specified major ICAs. Pooled proportion of ICA in RAA with RDA fetuses calculated with meta-analytic methodology, using the aforementioned random effect model yielded a proportion of 28.7% (95%CI 12.1–47.7; *p* = 0.449; Figure 2).

**Figure 3:**
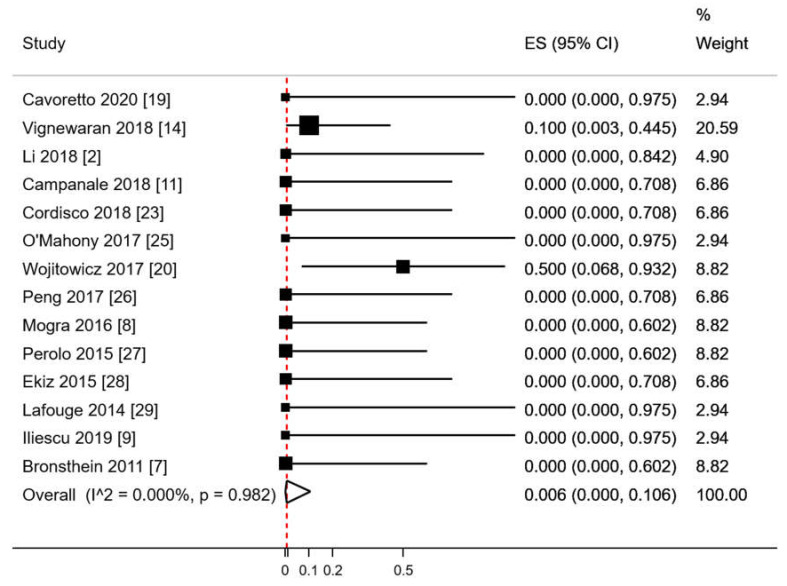
Pooled proportion of chromosomal anomalies in RAA/RDA fetuses who underwent karyotyping. ES: effect size. CI: confidence interval. Red dashed line describes the effect estimate found in the present analysis. I^2: I^2^ of Higgins.

The crude rate of chromosomal abnormalities among fetuses with a reported karyotype was 7% (3/44). The only chromosomal anomaly that we found in fetuses with RAA/RDA was 22q11.2 microdeletion, of which 2 (66.7%) also had thymic hypoplasia. The pooled proportion of chromosomal abnormalities in RAA/RDA fetuses tested for karyotyping using a random effects model was of 0.6% (95%CI 0.0–10.6; *p* = 0.982; Figure 3).

Associated ECA were reported only in 4 of the 53 of cases with known neonatal outcome (crude proportion 7.5%), of which none had major anomalies (cleft palate; dolichocephaly; persistent right umbilical vein). Specifically, one had a bilateral ductal arch and a right umbilical vein [21] another was a DiGeorge syndrome with left pulmonary artery stenosis and cleft palate [14] and two were dolichocephalic fetuses with dysmorphic features observed at birth (one of the latter had DiGeorge syndrome) [20].

**Figure 4:**
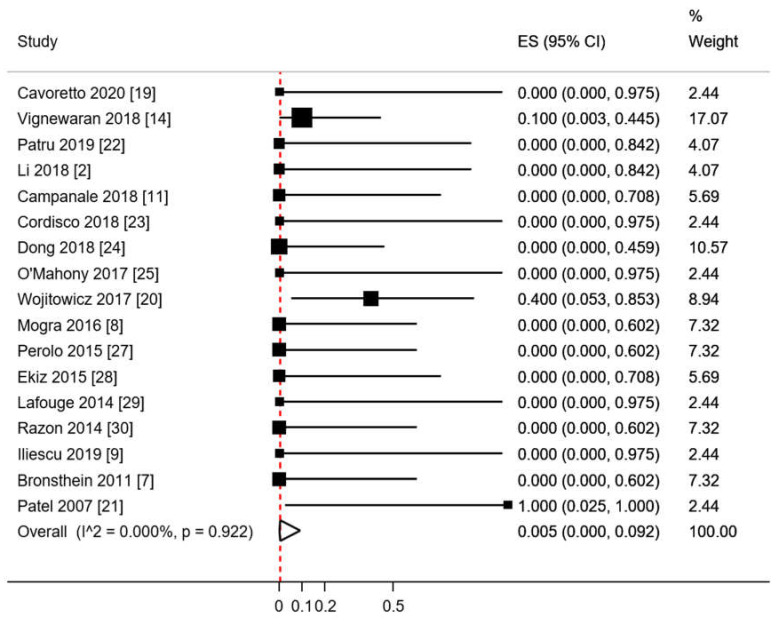
Pooled proportion of extracardiac abnormalities in RAA/RDA fetuses. ES: effect size. CI: confidence interval. Red dashed line describes the effect estimate found in the present analysis. I^2: I^2^ of Higgins.

The pooled proportion of ECA in RAA with RDA fetuses calculated with metanalytic methodology, using a random effects model was 0.5% (95%CI 0.0–9.2; *p* = 0.922; Figure 4).

**Figure 5:**
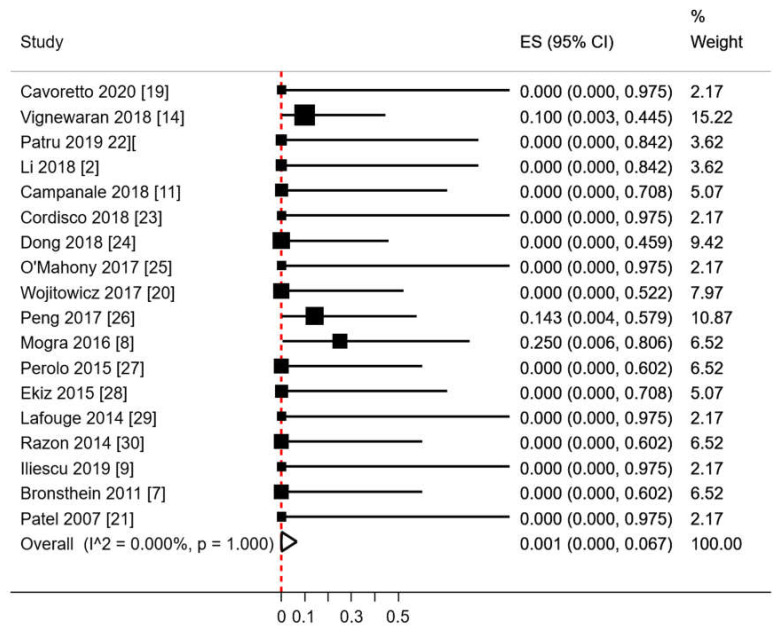
Pooled proportion of aberrant left subclavian artery in RAA/RDA fetuses. ES: effect size. CI: confidence interval. Red dashed line describes the effect estimate found in the present analysis. I^2: I^2^ of Higgins.

ALSA was only observed in 5% of RAA/RDA fetuses (3/60). The pooled proportion of ALSA in RAA with RDA fetuses calculated with metanalytic methodology, using a random effect model was of 0.1% (95%CI 0.0–6.7; *p* = 1.000; Figure 5).

Almost all prenatally detected RAA/RDA cases were confirmed on postnatal assessment (based on available postnatal outcomes) except for one case, which was postnatally diagnosed with RAA and bilateral DA. The latter is the only case that underwent post-natal cardiac surgery. Syndromic features were reported postnatally in two cases (one case had hypertelorism, a wide nasal bridge, an absent right ear, and an epibulbar dermoid) [21]; while the other had unspecified syndromic features and was asymptomatic at 8 months [20]). A favorable neonatal outcome was reported in 49 cases. Three cases developed neonatal respiratory symptoms; one of them was identified with ALSA forming a vascular ring and two cases were probably due to bronchial compression or anomalous development. 

#### 3.2.2. Secondary Analyses: Outcomes in RAA/RDA versus RAA/LDA

**Figure 6:**
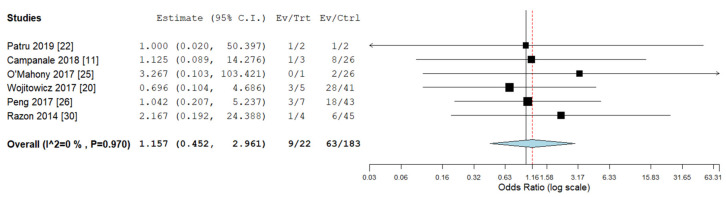
Pooled odd ratio (OR) for intracardiac abnormalities in right aortic arch with concomitant right ductal arch and right aortic arch with concomitant left ductal arch. (black vertical line: OR = 1, red dashed line = estimated pooled OR in the present analysis). I^2: I^2^ of Higgins.

**Figure 7:**
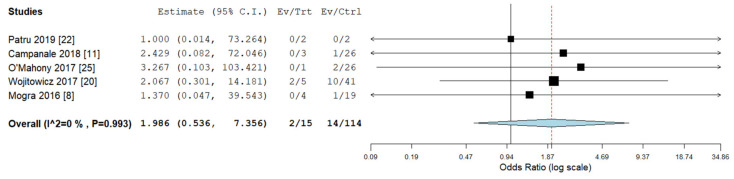
Pooled odd ratio (OR) for extracardiac abnormalities in right aortic arch with concomitant right ductal arch and right aortic arch with concomitant left ductal arch. (black vertical line: OR = 1, red dashed line = estimated pooled OR in the present analysis). I^2: I^2^ of Higgins.

**Figure 8:**
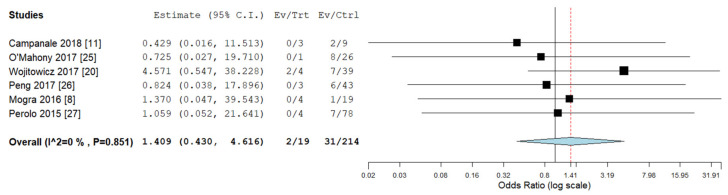
Pooled odd ratio (OR) for chromosomal abnormalities in right aortic arch with concomitant right ductal arch and right aortic arch with concomitant left ductal arch. (black vertical line: OR = 1, red dashed line = estimated pooled OR in the present analysis). I^2: I^2^ of Higgins.

**Figure 9:**
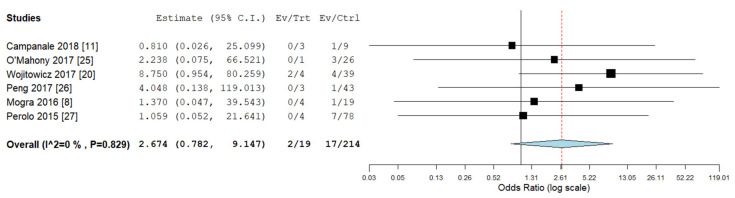
Pooled odd ratio (OR) for 22q11 microdeletion in right aortic arch with concomitant right ductal arch and right aortic arch with concomitant left ductal arch. (black vertical line: OR = 1, red dashed line = estimated pooled OR in the present analysis). I^2: I^2^ of Higgins.

**Figure 10:**
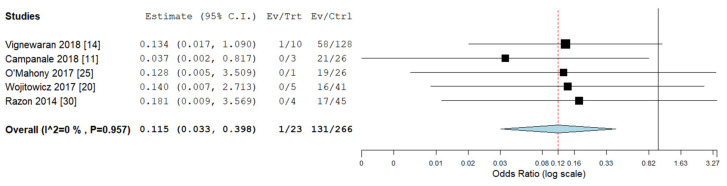
Pooled odd ratio (OR) for aberrant left subclavian artery in right aortic arch with concomitant right ductal arch and right aortic arch with concomitant left ductal arch. (black vertical line: OR = 1, red dashed line = estimated pooled OR in the present analysis). I^2: I^2^ of Higgins.

Pooled odds ratios (ORs) for ICA, ECA, chromosomal abnormalities, 22q11deletion, and ALSA were not significantly different between RAA/RDA compared to RAA/LDA fetuses collected from the same cohort (Figure 6, Figure 7, Figure 8, Figure 9 and Figure 10). 

#### 3.2.3. Other Results

Herein we describe an additional case from our experience added to the present review and recently presented [19].

A low-risk 38-year-old woman gravida 2 para 1 with no significant past medical history attended our hospital for routine pregnancy ultrasound scans. Her first-trimester scan was normal. cf-DNA screening for trisomy 13,18,21 resulted in low risk (fetal fraction 11.8%). During her routine anomaly scan at 20 weeks (Voluson E10, GE Healthcare, Zipf, Austria) equipped with 4–8 MHz multi-frequency convex transducer), a right aortic arch (RAA) and a right ductus arteriosus (RDA) were detected while assessing the three-vessels and trachea view (3VT). Fetal echocardiography confirmed a mirror-image aortic arch (AA) and a V-shaped appearance of the junction between the DA and AA on the right side of the trachea with normal intracardiac anatomy. The thymus was present. No extracardiac abnormalities were observed. Additional testing for 22q11.1 microdeletion was requested with normal results. Spontaneous delivery occurred at 39 weeks with normal weight and APGAR score. At the time of data collection, the newborn is 16 weeks old and asymptomatic. 

### 3.3. Risk of Bias Assessment

Table 1 includes the quality assessment performed using the NIH tool for the quality assessment of case study series. The main issues of the case series are related to small sample size, lack of adequate neonatal follow-up after delivery, and the lack of detail on single-case outcomes in case series.

## 4. Discussion

### 4.1. Summary of Key Findings

Using meta-analytic techniques, we found that a right aortic arch with right ductal arch (RAA/RDA) is associated with an approximate 30% risk for conotruncal CHDs and 1% risk for 22q11; two-thirds of 22q11 cases had concomitant thymic hypoplasia. No other chromosomal defects were described. The risks for ICA, ECA, 22q11 microdeletion, and ALSA are not substantially different in RAA with right or left arterial duct. The risks for other associated problems or need for surgery are rather low, as only one case (with double DA) underwent post-natal surgery.

### 4.2. Interpretation

The prenatal diagnosis of aortic arch abnormalities, such as RAA, has increased since the recent introduction of the three-vessel trachea view on routine US scan in 2015 (UK FASP guidelines [31]). The presence of RDA, on the other hand, is described more rarely. Some of the previous reports support the association between RDA/RAA and other ICA/ECA or Di George syndrome, [12] while others fail to describe associated abnormalities and/or chromosomal/genetic syndromes [7]. These controversial results are probably due both to the rarity of the condition and the differences in study designs and settings (lack of risk stratification in the examined populations and/or different expertise of diagnostic methods) [7,12,14].

It is important for sonographers to search for retrotracheal aberrant vessels in the context of any conotruncal abnormality, as the associated diagnosis of an aberrant subclavian artery may lead to tracheal obstruction/compression-related symptoms postnatally. However, we did not observe significantly different risk of ALSA in RAA with left or right ductal arch, which indicates that the counseling on post-natal respiratory symptoms should be similar regardless of the laterality of the ductus. While ARSA mainly related to trisomy 21, [32] ALSA was not associated with trisomy 21 in this context, but only to 22q11 microdeletion.

We observed that genetic testing in our study sample was only available for 44 out of 60 cases (73%). This was mainly due to parents’ decision; however, there is no evidence that the risk of chromosomal defects was discussed in all included cases. DiGeorge syndrome was the only genetic defect reported in our RAA/RDA fetuses. Obviously, without genetic testing on all patients, the prevalence of 22q11 microdeletion is likely to be underestimated. We believe that it would be good practice to offer genetic testing for this abnormality in all cases with RAA, regardless of the ductal arch laterality. The role of non-invasive prenatal testing might be selectively discussed in cases without associated anomalies, including analysis for 22q11 microdeletion. However, fetuses with associated extracardiac or intracardiac malformations would require invasive testing with chromosomal microarray.

Fetal growth in fetuses with CHDs may be impaired, particularly in the cyanotic subgroup [33] However, in our study, the prevalence of fetal growth restriction and small for gestational age was not different from the general population.

Technical expertise is required to diagnose cardiac defects before birth, and further implementation of ISUOG guidelines with the use of the three vessel and trachea view would contribute to increased detection of aortic arch abnormalities. In the same view, it will be possible to confirm the presence of a well-identifiable thymus, which is a reassuring finding in patients with RAA/RDA.

To date, there is no evidence highlighting the need for different counseling between isolated RAA/RDA and isolated RAA/LDA fetuses, as shown in our pooled odds ratio analysis. 

We are aware of the limitations of this study, the main one being the relatively small number and sample size of included studies, differences in reporting neonatal outcomes, and referral bias. Moreover, the examined articles showed heterogeneity in evaluated outcomes, with different periods of follow up after birth. The possibility of a consistent and thorough analysis is limited, which might affect the applicability of these results to the general population. 

## 5. Conclusions

RAA with RDA is associated with a high risk of intracardiac abnormalities (about 30%) and 22q11 microdeletion risk of around 1%. Other risks appear similar regardless of the laterality of the ductal arch. Therefore, in isolated cases, we recommend reassurance, particularly when the thymus and karyotype are normal.

## Figures and Tables

**Table 1:** Study characteristics, outcomes, and quality score.

Study	Country	RAA RDA	RAA LDA	GA Diagnosis of RAA + RDA (Mean)	Outcomes Observed	GRADE AHQR Standards
Cavoretto (2020) [19]	Italy	1	NA	20	ICA, ECA and CA	fair
Patru (2019) [22]	Romania	2	2	Second trimester	ICA, ECA and CA	fair
Vigneswaran (2018) [14]	UK	10	NS	median 21 (range 11–36)	ICA, ECA and CA	good
Li (2019) [2]	China	2	0	NA	ICA, ECA and CA	good
Campanale (2018) [11]	Italy	3	26	mean 26 ± 5	ICA, ECA and CA	fair
Cordisco (2018) [23]	Italy	1	NA	25	ICA, ECA and CA (bilateral DA)	fair
Dong (2018) [24]	China	6	0	28.2 (range 24–35)	ICA, ECA CA = 0	good
O’Mahony (2017) [25]	Australia	1	29	20	ICA, ECA and CA	good
Wòjtowicz (2017) [20]	Poland	5	41	24.6 (range 20–31)	ICA, ECA and CA	good
Peng (2017) [26]	China	7	43	mean 24	ICA, and CA ECA = 0	fair
Mogra (2016) [8]	Australia	4	19	mean 19	ICA, ECA and CA	fair
Perolo (2016) [27]	Italy	4	NA	mean 20.9 ± 3	ICA, ECA and CA	fair
Ekiz (2015) [28]	Turkey	3	0	31 + 6; 22 + 4; NA	ICA, ECA and CA	good
Lafouge (2014) [29]	France	1	NA	11 + 6	ICA, ECA and CA	fair
Razon (2014) [30]	Israel	4	45	mean 22.7	ICA and ECA CA = 0	fair
Iliescu (2012) [9]	Romania	1	NA	first trimester	ICA, ECA and CA	fair
Bronsthein (2011) [7]	Israel	4	0	89% between 14–16 weeks	ICA and ECA CA = 0	good
Patel (2007) [21]	USA	1	NA	25	ICA, ECA and CA	fair

CA: chromosomal abnormalities; ICA: intra-cardiac abnormalities; ECA: extracardiac abnormalities, NA: not available, NS: not separate data. RAA: right aortic arch. RDA: right ductal arch. LDA: left ductal arch. GA: gestational age. GRADE AHQR: Agency for Healthcare Research and Quality.

## Abbreviations

ALSA	aberrant left subclavian artery
DA	ductus arteriosus
ECA	extra-cardiac abnormalities
ICA	intra-cardiac abnormalities
LAA	left sided aortic arch
LDA:	left sided ductal arch
ISUOG	International Society of Ultrasound in Obstetrics and Gynecology (ISUOG)
NICE	National Institute for Health and Care Excellence (United Kingdom)
NIH	National Institute of Health (United States of America)
RAA	right sided aortic arch
RDA	right sided ductal arch
RAA/RDA	right aortic arch with concomitant right ductal arch
